# Optical Coherence Tomography to Assess Neurodegeneration in Phenylalanine Hydroxylase Deficiency

**DOI:** 10.3389/fneur.2021.780624

**Published:** 2021-12-10

**Authors:** Amelie S. Lotz-Havla, Katharina Weiß, Katharina Schiergens, Stephanie Regenauer-Vandewiele, Klaus G. Parhofer, Tara Christmann, Luise Böhm, Joachim Havla, Esther M. Maier

**Affiliations:** ^1^Dr. von Hauner Children's Hospital, LMU University Hospital, Ludwig-Maximilians-Universität München, Munich, Germany; ^2^Medical Department IV - Grosshadern, LMU University Hospital, Ludwig-Maximilians-Universität München, Munich, Germany; ^3^Institute of Clinical Neuroimmunology, LMU University Hospital, Ludwig-Maximilians-Universität München, Munich, Germany; ^4^Data Integration for Future Medicine (DIFUTURE) Consortium, Ludwig-Maximilians-Universität München, Munich, Germany

**Keywords:** phenylketonuria, PKU, phenylalanine hydroxylase deficiency, optical coherence tomography, OCT, retinal neuroaxonal degeneration, neurodegeneration

## Abstract

In phenylalanine hydroxylase (PAH) deficiency, an easily feasible method to access the progression of neurodegeneration is warranted to contribute to current discussions on treatment indications and targets. The objective of the present study was to investigate whether optical coherence tomography (OCT) measures as markers of neurodegeneration differ between patients with PAH deficiency and healthy controls (HCs) according to phenotype and metabolic control. In this single-center cross-sectional study, 92 patients with different phenotypes of PAH deficiency [PAH deficiency not requiring treatment, early treated phenylketonuria (ETPKU), and late-diagnosed phenylketonuria (PKU)] compared with 76 HCs were examined using spectral-domain OCT. Indices of phenylalanine elevation and variability were correlated with OCT parameters. Late-diagnosed PKU patients showed reduced peripapillary retinal nerve fiber layer (pRNFL) thickness and combined ganglion cell and inner plexiform layer (GCIPL) volume. Adult ETPKU patients were found to have lower GCIPL volume (*p* = 0.016), which correlated with the indices of phenylalanine control. In pediatric ETPKU patients with poor metabolic control, pRNFL was significantly reduced (*p* = 0.004). Patients with PAH deficiency not requiring treatment did not exhibit retinal degeneration. Inner nuclear layer (INL) was significantly increased in the pediatric ETPKU patients, driven by those with current poor metabolic control (*p* = 0.006). Our data provide evidence of retinal neuroaxonal degeneration and INL swelling, depending on the phenotype, current age, and metabolic control. These findings suggest that OCT is suitable to investigate neurodegeneration in PKU and we propose OCT as a sensitive, reliable, safe, low-burden, and low-cost examination for future multicenter studies.

## Introduction

Phenylalanine hydroxylase (PAH) deficiency (OMIM #261600) is caused by autosomal recessive variants in the phenylalanine hydroxylase (*PAH*) gene and leads to an impaired degradation of the amino acid phenylalanine (Phe) to tyrosine and, as a consequence, to elevated concentrations of Phe in blood (1). According to current recommendations, PAH deficiency is classified as PAH deficiency not requiring treatment and PAH deficiency requiring treatment (hereinafter referred to as phenylketonuria, PKU) (2).

If untreated, PKU leads to severe brain damage with intellectual disability, seizures, and spasticity (3). This severe clinical phenotype is avoided by the introduction of newborn screening enabling an early initiation of dietary therapy to lower Phe concentrations in blood (4). Lately, the approvals of BH_4_ (Kuvan® sapropterin dihydrochloride), the natural cofactor of PAH, for BH_4_-responsive patients and pegvaliase-pqpz (Palynziq®), a recombinant phenylalanine ammonia lyase, for adolescent and adult patients have expanded the treatment options for PKU, and thereby reduced the burden of a strict low-Phe diet for at least some of the patients (1, 5–9).

Despite these advances, data regarding the optimal treatment targets for PKU patients are insufficient leading to different treatment recommendations worldwide (2, 10). Additionally, there is only consensus that patients with Phe concentrations above 600 μmol/L do require treatment (2), and individuals with Phe concentrations below 360 μmol/L do not (2, 11). However, it remains under debate whether treatment is indicated in individuals with Phe concentrations between 360 and 600 μmol/L (2, 12–16). Neither neurocognitive nor MRI outcome studies have yet contributed to a clear decision on these issues.

White matter lesions (WMLs) have described as a marker of disease progression in PKU (17, 18). The extent of WMLs has been shown to be associated with the patient's age and metabolic control (17, 19–22). In untreated PKU patients, hypomyelination has been attributed to WMLs (17, 23, 24). In early treated PKU patients (ETPKU), WMLs are likely to reflect intramyelinic edema (17, 25) that can be reversed with re-adherence to a strict low-Phe diet (25, 26).

To address these unanswered questions, a monitoring test to assess the progression of neurodegeneration that is safe, low burden, and low-cost for the patients would be helpful.

Optical coherence tomography (OCT) is a non-invasive examination technique of the retina that allows the assessment of retinal neuroaxonal degeneration (27). OCT measurements have been identified as marker of disease progression in different (28–31) and neurodegenerative disorders (32, 33), as well as in metabolic neurodegenerative diseases, such as Wilson disease (34) and Niemann-Pick disease type C (35, 36).

Only recently, conflicting results of OCT studies on ETPKU cohorts have been described (37–40). Hopf et al. did not find any pathologies in the OCT measurements of the macula and optic nerve head in 10 pediatric and 9 adult PKU patients (37), whereas two other studies found evidence of retinal axonal degeneration in early treated pediatric (38) and adult PKU patients (39), as well as retinal neuronal degeneration in the early treated adult PKU patients (40).

To test the hypothesis that OCT is suitable to detect neurodegeneration in PAH deficiency, the present study investigated neuroaxonal retinal degeneration in patients with PAH deficiency according to phenotype and metabolic control. For this, (i) a large pediatric and adult cohort covering the entire phenotypic spectrum, from PAH deficiency not requiring treatment, over ETPKU to severely affected late-diagnosed PKU patients, was analyzed in comparison with the healthy controls (HCs), and (ii) the correlation of OCT measures with Phe elevation and variation was assessed. Beyond this, given the presumed WMLs pathology in ETPKU, the retinal correlate of cerebral intramyelinic edema was examined by analysis of the inner nuclear layer.

## Materials and Methods

### Study Population

All registered patients who were diagnosed with PAH deficiency during neonatal or selective screening, and who were under regular care at the metabolic center of the LMU Hospital, Ludwig-Maximilians-University in Munich, Germany were invited to participate in this study. The inclusion criteria were confirmed PAH deficiency and age 6 years and older. The exclusion criteria were: (i) ocular comorbidities potentially confounding interpretation of OCT results (>±5.5 diopters of spherical equivalent, >±3 diopters of astigmatism, history of ocular disease, e.g., macular degeneration, glaucoma, and intracranial hypertension), (ii) history of systemic disease known to affect the retina [e.g., diabetes (41, 42)], (iii) history of any neurological disease unrelated to PAH deficiency, (iv) prematurity <36 weeks of gestational age (43), (v) current pregnancy (44), and (vi) interfering medical treatment. The exclusion criteria were identified based on the medical history of patients.

In this study, 150 eligible patients were prospectively identified and approached about the possibility of study participation. Ninety-five patients decided to participate, and of these 92 patients could be prospectively included in the study between October 2018 and January 2021 (as shown in Table 1). Three patients could not be included due to exclusion criteria (history of bilateral chorioretinitis, glaucoma, and >±5.5 diopters of spherical equivalent).

**Table 1:** Demographic information of cohorts and phenylalanine indices of the phenylalanine hydroxylase (PAH) deficient patient groups.

	**HC**		**PAH deficient patients**					
			**Not requiring treatment**		**ETPKU**		**PKU, late diagnosed**	
	**(*****N*** **=** **76)**		**(*****N*** **=** **18)**		**(*****N*** **=** **70)**		**(*****N*** **=** **4)**	
	**Mean**	**SD**	**Mean**	**SD**	**Mean**	**SD**	**Mean**	**SD**
Age in years (range)	33 (7–59)	15	19 (7–50)	12	21 (7–54)	11	47 (20–59)	18
Gender f/m	50/26		14/4		41/29		3/1	
**Blood Phe [μmol/l]**								
**Childhood (0–10 years)**								
IDC			238	101	259	95	192	102
Average of yearly SD			55	25	155	53	226	64
**Adolescence (11–16 years)**								
IDC			269	128	498	208	586	354
Average of yearly SD			49	28	157	60	146	25
**Adulthood (17 years** **+)**								
IDC			269	106	662	313	645	261
Average of yearly SD			55	44	145	66	196	35
**Lifetime**								
IDC			238	88	405	212	581	276
Mean Phe			232	79	382	184	534	248
Mean exposure			−0.99	1.11	−0.01	1.38	3.90	2.94
Average of yearly SD			55	29	150	48	199	29
SD Phe			68	31	249	105	290	59
SD exposure			−1.55	0.96	0.30	1.40	3.00	2.10
**Current Phe**			n.a.	n.a.	552	404	948	626

*IDC, average of yearly median phenylalanine levels; Phe, phenylalanine; HC, healthy control; PAH, phenylalanine hydroxylase; PKU, phenylketonuria; n.a., not applicable*.

Patients with PAH deficiency were classified as follows: patients requiring treatment (*N* = 74) and patients not requiring treatment (*N* = 18) (2, 45). The indication for treatment was based on the German recommendations at the time of diagnosis (46), i.e., therapy was initiated when Phe concentrations in the untreated patients exceeded 600 μmol/L. Patients with PAH deficiency requiring treatment were further divided into ETPKU and late-diagnosed PKU patients. The ETPKU group (*N* = 70) comprised patients who were diagnosed by neonatal screening. The group of late-diagnosed PKU patients (*N* = 4, age at diagnosis mean 22 months, SD 5.8 months, range 12–30 months) included patients who did not undergo newborn screening and were thus diagnosed and treated after the onset of symptoms. Seventy-six HCs matched for age and gender of the patients were also included in the study.

The study was performed in accordance with the Helsinki II Declaration and approved by the ethics committee of the Ludwig-Maximilians-University of Munich, Medical Faculty (part of project no 18-256). All the participants and/or their legal representatives gave written informed consent.

### Spectral-Domain OCT

Optical coherence tomography examination was performed using a SD-OCT (Spectralis, Heidelberg Engineering, Heidelberg, Germany) with automatic real-time (ART) function for image averaging. Data are reported for peripapillary retinal nerve fiber layer thickness (pRNFL) to assess axonal degeneration. Total macular volume (TMV), volumes of combined ganglion cell and inner plexiform layer (GCIPL = GCL + IPL), and inner retinal layer (IRL = GCL + IPL + mRNFL) were assessed as markers for neuronal degeneration. Data for inner nuclear layer (INL) were evaluated to detect edema-related retinal changes. Calculation of macular layers is given for a 3 mm diameter cylinder around the fovea from a macular volume scan (20° × 20°, 25 vertical B-scans, ART ≤ 49). The pRNFL was measured with an activated eye tracker using 3.4 mm ring scans around the optic nerve (12°, 1,536 A-scans, ART ≤ 100). Segmentation of all the layers was performed semi-automatically using software provided by the OCT manufacturer (Eye Explorer 1.9.10.0 with viewing module 6.3.4.0, Heidelberg Engineering, Heidelberg, Germany). All the scans were checked for sufficient quality and segmentation errors and corrected, if necessary. OCT data are reported according to the APOSTEL and OSCAR-Ib recommendations (47–49). Data were analyzed separately for the patients up to 17 years of age and adults.

### Indices of Metabolic Control

For all PAH deficient patients, comprehensive Phe monitoring data were available. Limited data were available for the patients who were treated at other metabolic centers in childhood (*N* = 9) or had poor adherence in adulthood (*N* = 2). To calculate the indices of Phe control, we combined previously proposed approaches (19, 50, 51). We averaged Phe control in the following age bands: childhood 0–10 years of age, adolescence 11–16 years of age, adulthood 17 years of age to present, and lifetime. For each age band, we considered the two measures Phe average and Phe variation (50). The Phe average was calculated by averaging the yearly median Phe levels (IDC). The Phe variation was calculated by averaging the SD for each year (50). We furthermore calculated the mean (mean Phe) and SD (SD Phe) of all available Phe levels for each patient (19). To take into account the duration (i.e., years) and accumulative effects of exposure to elevations and variability in Phe, we calculated mean exposure and SD exposure as previously described (19). Furthermore, we considered the current Phe level determined at the time of OCT examination.

### Statistical Analyses

The statistical analyses were performed using SPSS Statistics 26 (IBM, NY, USA) by the authors (ASL-H). Comparison of demographic data between the patient and control group was analyzed by using the chi-square test. Both eyes of each subject were included in the analysis as statistically dependent duplicates. Data were analyzed for normal distribution using a Shapiro–Wilk test and a Q-Q plot. To compare the PKU patients disease with controls, an unpaired *t*-test was used. To correct for multiple comparisons in the subgroup analysis, ANOVA with the Games-Howell *post-hoc* test was applied. The *p-*values below 0.05 were considered significant. A Pearson correlation analysis was performed to analyze the linear correlations of OCT parameters and indices of Phe control. Curve fitting using regression analysis and visual inspection of scatter plots after Locally Weighted Scatterplot Smoothing (LOESS) smoothing was applied to assess the relationship of variables in a correlation analysis. The subjects with missing data were excluded from the respective analysis. For two reasons, the correlation analyses were performed only for the group of adults aged 18–33 years (*N* = 32): (i) as expected from the literature (52), GCIPL volume was not associated with age in this cohort, and (ii) most comprehensive documentation of Phe levels was available.

## Results

### Patient Characteristics and Indices of Metabolic Control

Table 1 shows demographic data of age, gender, and Phe control. On average, the ETPKU patients and the late-diagnosed PKU patients showed a good Phe control in childhood and adolescence (2). In adult age, IDC was slightly above the recommendation of <600 μmol/L (2) (ETPKU 662 μmol/L and late-diagnosed PKU 645 μmol/L). Variability in Phe was largely consistent across all age ranges.

The ETPKU cohort was further subdivided based on the average Phe levels as follows: ETPKU patients whose Phe levels were always within recommendations (ETPKU1: *N* = 41, IDC childhood 227 ± 52 μmol/L, IDC adolescence 358 ± 105 μmol/L, and IDC adulthood 416 ± 147 μmol/L) and ETPKU patients whose Phe levels were outside recommendations in childhood, adolescence, and/or adulthood (ETPKU2: *N* = 29, IDC childhood 305 ± 123 μmol/L, IDC adolescence 644 ± 188 μmol/L, and IDC adulthood 838 ± 280 μmol/L) (2).

Among 70 ETPKU patients, 36 (51%) were BH_4_ responsive and treated with BH_4_, alone or in combination with dietary therapy. Of note, BH_4_ responsive patients showed significantly lower Phe concentrations after the relaxation of treatment suggested in adolescence as compared with BH_4_ non-responsive patients (Table 2). In addition, the variability in Phe was significantly lower at all ages (Table 2).

**Table 2:** Phenylalanine indices of BH_4_ responsive and non-responsive ETPKU patients.

	**BH**_**4**_ **responder**		**BH**_**4**_ **non-responder**		
	**(*****N*** **=** **36)**		**(*****N*** **=** **34)**		
	**Mean**	**SD**	**Mean**	**SD**	***p***
**Blood Phe [μmol/l]**				
**Childhood (0–10 years)**				
IDC	250	96	269	96	*0.431*
Average of yearly SD	131	43	182	50	0.000^*^
**Adolescence (11–16 years)**				
IDC	429	182	565	214	0.018^*^
Average of yearly SD	131	55	180	54	0.002^*^
**Adulthood (17 years** **+)**				
IDC	551	253	780	332	0.017^*^
Average of yearly SD	114	52	176	66	0.002^*^
**Lifetime**				
IDC	331	144	482	244	0.002^*^
Mean Phe	322	135	445	208	0.004^*^
Average of yearly SD	122	37	180	39	0.000^*^
SD Phe	194	94	307	84	0.000^*^

*Abbreviations: IDC, average of yearly median phenylalanine levels; Phe, phenylalanine*.

* *p < 0.05, p-values were calculated using the unpaired t-test*.

PAH deficient patients not requiring treatment treatment had average lifetime Phe concentrations below 360 μmol/L (Table 1). Only two patients assigned to this group had recurrent Phe concentrations between 360 and 600 μmol/L, all others had Phe concentrations <360 μmol/L in healthy state. The variability in Phe was significantly lower in PAH deficient patients not requiring treatment compared with those requiring treatment (*p* < 0.05 for all Phe indices).

### Retinal Neuroaxonal Degeneration in the Late-Diagnosed PKU Patients

**Figure 1:**
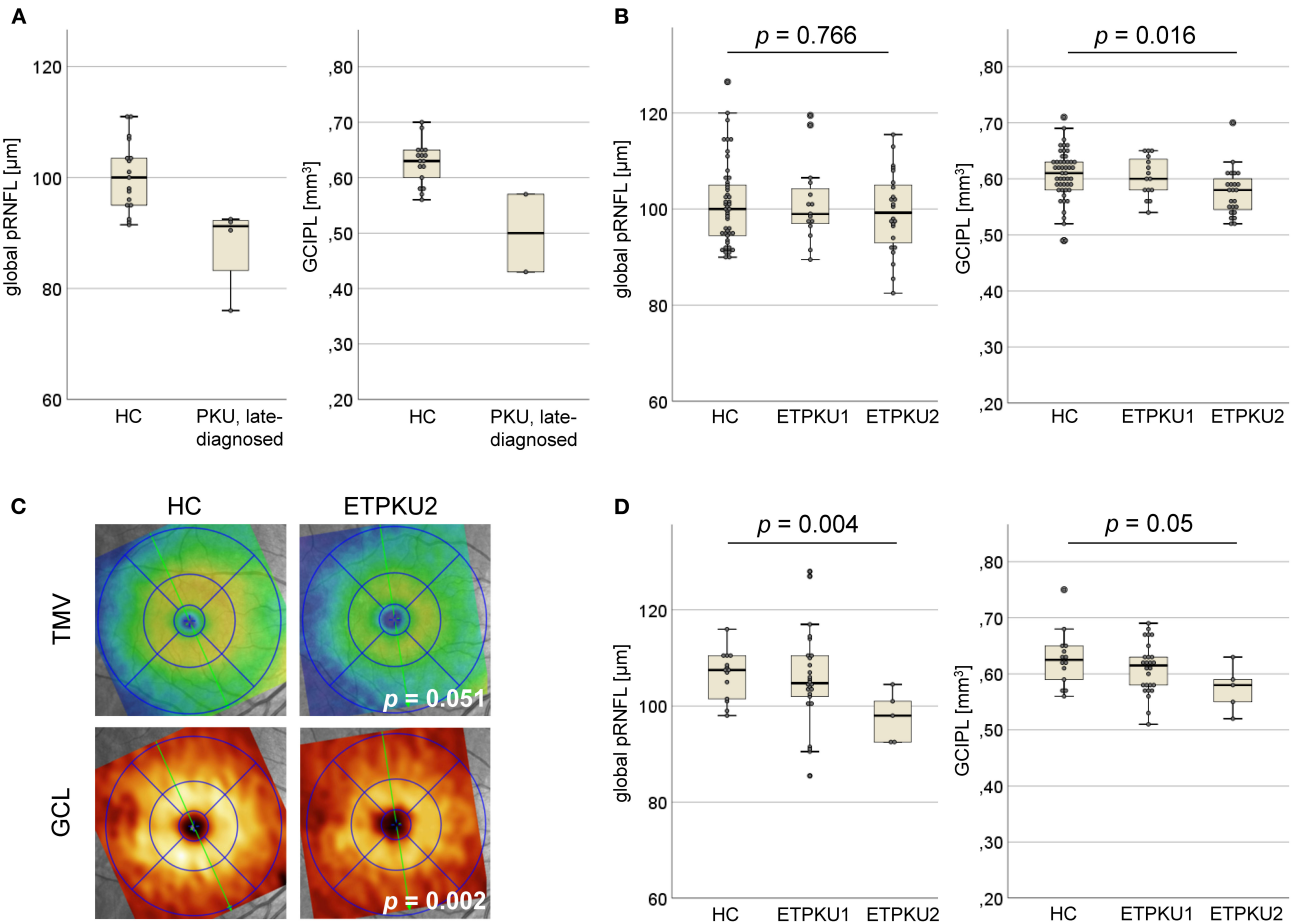
Axonal and neuronal degeneration in phenylketonuria (PKU). **(A)** Global peripapillary retinal nerve fiber layer (pRNFL) thickness and ganglion cell and inner plexiform layer (GCIPL) volume in the late-diagnosed PKU patients compared with HCs (*N* = 42). **(B)** Global pRNFL thickness and GCIPL volume in early treated phenylketonuria (ETPKU) ≥ 18 years of age compared with HCs (*N* = 49). Based on average of yearly median phenylalanine levels (IDC) (as shown in Table 1) in childhood, adolescence, and adulthood, the metabolic control of patients was analyzed and ETPKU were grouped according to adherence to European Union (EU) guidelines (ETPKU1; IDC in childhood <360 and <600 μmol/L after that, *N* = 15, ETPKU2; IDC in childhood >360 μmol/L and/or >600 μmol/L after that, *N* = 24). **(C)** Total macular volume (TMV) and ganglion cell layer (GCL) volume in ETPKU2 ≥ 18 years of age. Depicted is the heat map of the examination of a single patient with ETPKU2 and the corresponding HC, with a coding for TMV (blue tones; low volume, yellow/red tones; high volume) and GCL (brown tones; low volume, yellow tones; high volume). **(D)** Global pRNFL thickness and GCIPL volume in HCs (*N* = 14), ETPKU1 (*N* = 26), and ETPKU2 (*N* = 5) from 6 to 17 years of age. The *p*-values are given for comparisons between the ETPKU2 cohort and HCs.

Global pRNFL thickness was significantly reduced in the late-diagnosed PKU patients compared with age and sex matched HCs (mean ± SD 88 ± 7.9 vs. 100 ± 6.4 μm) (Figure 1A).

Due to the intellectual disability, two of the patients showed poor persistence during the OCT examination. Thus, performing a complete macular scan was possible only in the remaining two patients. In them, the volume of GCIPL was reduced compared with HCs (mean ± SD 0.5 ± 0.1 vs. 0.63 ± 0.04 mm^3^) (Figure 1A).

### Retinal Neuroaxonal Degeneration in the ETPKU Patients

Spectral-domain OCT studies were performed in 39 adult and 31 pediatric ETPKU patients.

The adult ETPKU patients showed a significant reduction in GCIPL volume compared with HCs (mean ± SD 0.59 ± 0.04 vs. 0.61 ± 0.05 mm^3^, *p* = 0.035). No significant differences were observed for the other macular layers and global pRNFL thickness (ETPKU vs. HCs: mean ± SD 100.1 ± 8.3 vs. 100.8 ± 8.7 μm, *p* = 0.678), even when only the temporal quadrant was considered (ETPKU vs. HCs: mean ± SD 70.6 ± 10.7 vs. 73.2 ± 15.5 μm, *p* = 0.375).

As a next step, data from BH_4_ responsive and non-responsive ETPKU patients were analyzed separately. BH_4_ non-responsive patients showed a significant reduction in GCIPL and IRL volume compared with HCs (Table 3). Again, no significant difference was observed for global and single quadrant pRNFL thickness. In BH_4_ responsive ETPKU patients, no significant alterations in any of the axonal or neuronal retinal layers were found (Table 3). Of note, BH_4_ non-responsive patients had significantly higher Phe levels and variations (Table 2).

**Table 3:** OCT findings related to phenotype of PAH deficient patients diagnosed within the neonatal period by newborn screening.

	**HC**		**PAH deficiency**, **not requiring treatment**			**PAH deficiency**, **requiring treatment**					
						**BH**_**4**_ **responder**			**BH**_**4**_ **non-responder**		
**age ≥ 18 years**	**(*****N*** **=** **49)**		**(*****N*** **=** **9)**			**(*****N*** **=** **20)**			**(*****N*** **=** **19)**		
	**Mean**	**SD**	**Mean**	**SD**	***p***	**Mean**	**SD**	***p***	**Mean**	**SD**	***p***
global pRNFL[μm]	100.83	8.70	103.83	5.18	*0.356*	101.57	8.53	*0.942*	98.47	8.02	*0.545*
TMV [mm^3^]	2.16	0.11	2.14	0.08	*0.934*	2.15	0.11	*0.938*	2.10	0.08	*0.123*
GCIPL [mm^3^]	0.61	0.05	0.60	0.04	*0.843*	0.60	0.05	*0.592*	0.58	0.03	0.021^*^
IRL [mm^3^]	1.64	0.09	1.62	0.08	*0.814*	1.63	0.10	*0.782*	1.59	0.07	0.033*
**age 6–17 years**	**(*****N*** **=** **14)**		**(*****N*** **=** **9)**			**(*****N*** **=** **16)**			**(*****N*** **=** **15)**		
	**Mean**	**SD**	**Mean**	**SD**	***p***	**Mean**	**SD**	***p***	**Mean**	**SD**	***p***
global pRNFL [μm]	106.46	5.06	105.17	9.35	*0.923*	105.50	10.12	*0.940*	103.20	9.46	*0.483*
TMV [mm^3^]	2.16	0.08	2.14	0.07	*0.905*	2.18	0.14	*0.919*	2.12	0.10	*0.610*
GCIPL [mm^3^]	0.63	0.05	0.60	0.02	*0.280*	0.61	0.05	*0.663*	0.60	0.05	*0.233*
IRL [mm^3^]	1.63	0.08	1.62	0.06	*0.892*	1.66	0.13	*0.815*	1.61	0.10	*0.787*

*pRNFL, peripapillary retinal nerve fiber layer; TMV, total macular volume; GCIPL, ganglion cell and inner plexiform layer; IRL, inner retinal layer; HC, healthy control; PAH, phenylalanine hydroxylase*.

* *p < 0.05, comparison analysis was performed by Anova and Games-Howell post-hoc test, the p-values are given in comparison to age-matched HCs*.

To investigate whether the observed differences were connected to higher average Phe levels, we compared OCT parameters of the ETPKU1 and ETPKU2 patients to HCs. At this, the ETPKU2, but not ETPKU1 patients, showed a significantly reduced GCIPL volume compared with HCs (mean ± SD 0.58 ± 0.04 vs. 0.60 ± 0.05 mm^3^) (Figures 1B,C). This finding was more pronounced, when analyzing the BH_4_ non-responsive patients only (mean ± SD 0.56 ± 0.24 mm^3^, *p* = 0.003).

Consistent with the reduced GCIPL volume, we also observed a significantly reduced IRL volume in the ETPKU2 cohort as compared with HCs (mean ± SD 1.58 ± 0.09 vs. 1.64 ± 0.09 mm^3^, *p* = 0.007). Although differences did not reach the level of significance (ETPKU2 vs. HCs: mean ± SD 2.1 ± 0.09 vs. 2.2 ± 0.11 mm^3^, *p* = 0.051), we found TMV atrophy in individual patients as depicted in Figure 1C.

Again, there was no significant difference in global pRNFL thickness of ETPKU1 vs. ETPKU2 vs. HCs (mean ± SD 101 ± 8.4 vs. 99.3 ± 8.4 vs. 100.3 ± 8.1 μm) (Figure 1B). This was evident when considering all pRNFL sectors individually, as well as temporal pRNFL with the papillomacular bundle (PMD) (ETPKU1 vs. ETPKU2 vs. HCs: mean ± SD 72.7 ± 9.8 vs. 69.2 ± 11.2 vs. 73.2 ± 15.5 μm).

Analyzing the pediatric ETPKU patients revealed no significant difference in macular layers (mean ± SD; GCIPL 0.60 ± 0.05 mm^3^, TMV 2.14 ± 0.12 mm^3^, IRL 1.63 ± 0.11 mm^3^) and pRNFL thickness (mean ± SD 103 ± 10.0) as compared with HCs. Separate analysis for BH_4_ responsiveness also revealed no differences (Table 3).

However, assigning pediatric ETPKU patients to the subgroups based on average Phe levels, a significantly reduced global pRNFL thickness was found in the ETPKU2 group in comparison with HCs (mean ± SD 98 ± 5.3 vs. 107 ± 5.1 μm; *p* = 0.004) (Figure 1D). In addition, there was a trend of GCIPL reduction in ETPKU2 patients (ETPKU2 vs. HCs: mean ± SD 0.57 ± 0.04 vs. 0.63 ± 0.05 mm^3^) and it was notable that individual patients in the ETPKU1 group also had reduced pRNFL thicknesses and GCIPL volumes compared with the HC group (Figure 1D).

### OCT in the PAH Deficient Patients Not Requiring Treatment

This group of patients did not show differences in any of the OCT parameters analyzed compared with HCs (Table 3). Of note, two of these patients had recurrent Phe values between 360 and 600 μmol/L but did not show retinal neuroaxonal degeneration (pRNFL; mean ± SD 105 ± 9.5 μm, GCIPL; mean ± SD 0.59 ± 0.03 mm^3^).

### Correlation Analyses of GCIPL Volume and Metabolic Indices

**Figure 2:**
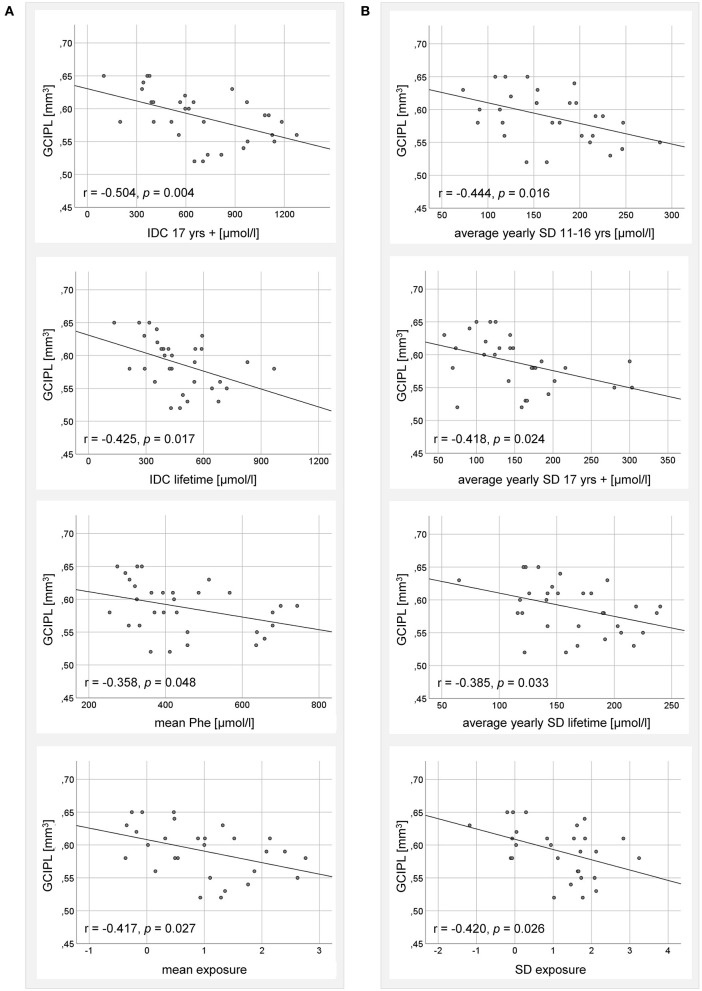
Correlation analysis between GCIPL volume of adult ETPKU patients 18–33 years of age (*N* = 32) and metabolic indices for **(A)** Phe elevation, and **(B)** Phe variability. IDC; average of yearly median Phe levels. The Pearson *r*- and *p*-values are indicated. The relationship of the correlating variables was linear, as depicted by the continuous line.

Looking at indices for Phe elevation, IDC in adulthood and lifetime, mean Phe and mean exposure was significantly negatively associated with GCIPL volume (Figure 2A). For IDC in childhood (*r* = 0.060, *p* = 0.753) and adolescence (*r* = −0.268, *p* = 0.152), no significant correlations were found.

Looking at indices for Phe variation, average yearly SD in adolescence, adulthood, and lifetime, as well as SD exposure were significantly negatively associated with GCIPL volume (Figure 2B). The average yearly SD in childhood (*r* = 0.026, *p* = 0.890) and the SD Phe (*r* = −0.347, *p* = 0.056) showed no significant correlations.

### Retinal Inner Nuclear Layer Volume in the ETPKU Patients

**Figure 3:**
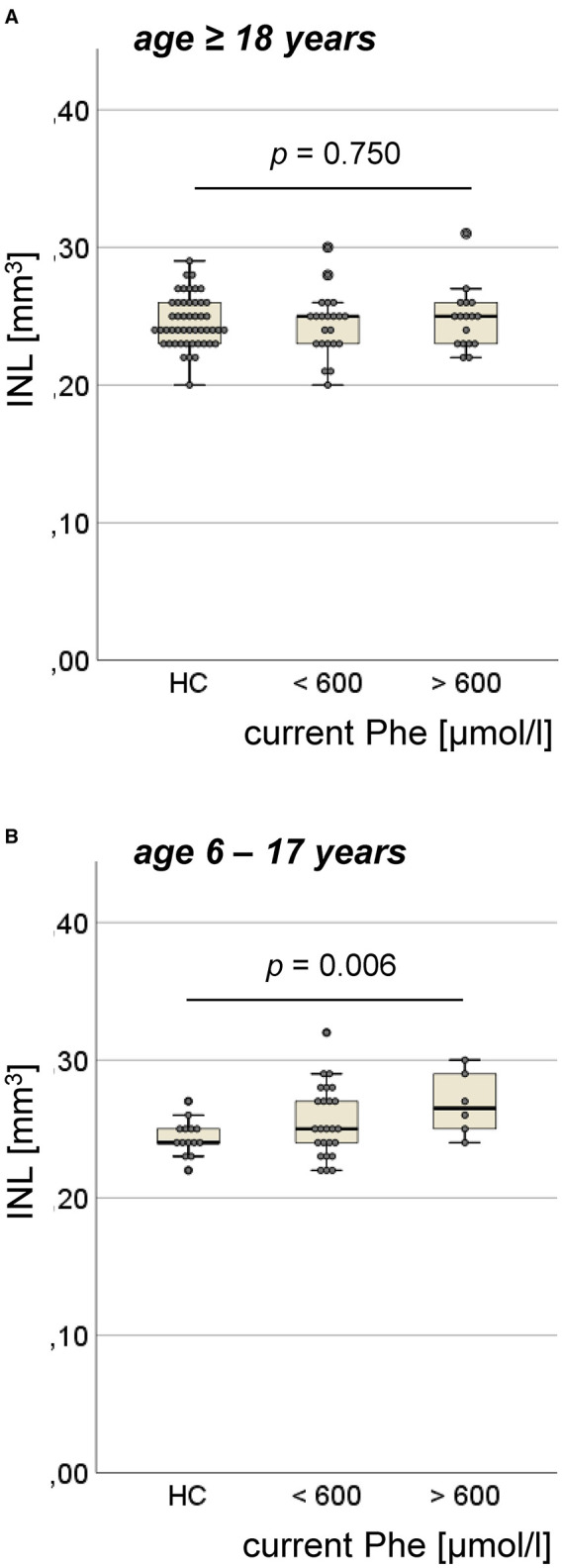
Volume of inner nuclear layer (INL) in the ETPKU patients. **(A)** ETPKU ≥ 18 years of age with current Phe level <600 μmol/L (*N* = 22) or >600 μmol/L (*N* = 17), and HCs (*N* = 49). **(B)** ETPKU 6–17 years of age with current Phe level <600 μmol/L (*N* = 25) or >600 μmol/L (*N* = 6), and HCs (*N* = 14). The *p*-values are given for comparison between ETPKU with current Phe level >600 μmol/L and HCs.

In adult ETPKU patients, no significant increase in INL volume compared with HCs was found (mean ± SD 0.25 ± 0.02 vs. 0.25 ± 0.02 mm^3^, *p* = 0.901), not even when analyzing only patients with a current Phe level of >600 μmol/L (Figure 3A). In general, INL volume did not correlate with the current Phe level (*r* = 0.12, *p* = 0.604).

In pediatric ETPKU patients, INL volume was significantly higher compared with HCs (mean ± SD 0.26 ± 0.03 vs. 0.24 ± 0.01 mm^3^, *p* = 0.017). This finding was more pronounced, when comparing only pediatric ETPKU patients with a current Phe level > 600 μmol/L to HCs (mean ± SD 0.27 ± 0.02 vs. 0.24 ± 0.01 mm^3^) (Figure 3B). However, we observed increased INL volume in a few patients with a current Phe level <600 μmol/L (Figure 3B) and there was no overall correlation of INL volume with the current Phe level (*r* = 0.109, *p* = 0.559).

## Discussion

To evaluate the potential of OCT parameters as markers of neurodegeneration in PAH deficiency, we performed spectral-domain OCT in pediatric and adult patients covering the complete phenotypic spectrum of PAH deficiency. Our major findings were (i) evidence of retinal neuroaxonal degeneration in late-diagnosed PKU patients, (ii) retinal neuroaxonal degeneration of varying degree related to age and metabolic control in ETPKU patients, (iii) no evidence of retinal degeneration in PAH deficient patients not requiring treatment, and (iv) increased INL volume in pediatric ETPKU patients.

In severely affected PKU patients, WMLs, most likely reflecting a lack of myelin formation, have been described (17, 23, 53). Consistent with this, the severely affected, late-diagnosed patients in our cohort showed reduced GCIPL volume and pRNFL thickness, suggesting retinal neuronal and axonal degeneration, respectively. These findings support the hypothesis of OCT parameters being potential markers of neurodegeneration in PAH deficiency.

The patients with PAH deficiency in whom Phe concentrations are found to be <600 μmol/L without treatment throughout life, represent the mildest manifestation of PAH deficiency and, thus, the opposite end of the phenotypic spectrum. These patients have been described to show no WMLs (12). Consistent with these findings, PAH deficient patients not requiring treatment in our cohort did not show any signs of retinal neuroaxonal degeneration. Since the majority of our patients in this group had Phe concentrations <360 μmol/L, our data mainly confirm the approach of not treating patients with baseline Phe concentrations below <360 μmol/L (2, 11). However, two of our patients had recurrent Phe levels between 360 and 600 μmol/L and did not show retinal neuroaxonal degeneration. With the limitation of two patients only, our data support the view of no indication for treatment in the patients with Phe concentrations below 600 μmol/L (12, 14). Of note, in both phenotypes, late-diagnosed PKU patients and PAH deficient patients not requiring treatment, the OCT studies have not been described so far.

In the ETPKU cohort, adult patients showed a significantly reduced GCIPL volume, indicating retinal neuronal degeneration. The observed effect was driven by the patients with poorer metabolic control, who also had a decreased IRL volume. This might also explain why BH_4_ non-responsive patients but not BH_4_ responsive patients showed significant neuronal degeneration. In patients who do not respond to BH_4_, good metabolic control is more difficult to achieve (5). This was also reflected by the significantly higher mean Phe levels and variability in BH_4_ non-responsive patients in our cohort. Accordingly, a correlation analysis demonstrated a negative association between GCIPL volume and indices of Phe elevation in adulthood or lifetime, and Phe variability from adolescence onward. Considering the good and consistent metabolic control of our adult cohort during childhood (IDC <360 μmol/L), a correlation analysis of GCIPL volume and childhood Phe indices was not meaningful. Based on these data one might hypothesize that retinal neuronal degeneration in adult ETPKU patients is triggered by increased and highly fluctuating Phe levels, whereas patients with good metabolic control do not show signs of retinal neuronal degeneration. Our observation of retinal neuronal degeneration in ETPKU is in line with the recent study of Serfozo et al. demonstrating significant IRL thinning in ETPKU compared with HCs (39, 40). However, this study described correlations to be found solely between the parafoveal IRL thickness and Phe levels within the last 10 years and therefore concluded no overall correlation between ganglion cell complex layer thickness and metabolic control (40). The discrepancies to our correlation analysis may arise from the OCT parameters analyzed and different OCT protocols used. Based on the experience of other diseases, GCIPL has been shown to be a reliable and sensitive marker of neurodegeneration (28, 54, 55). We therefore propose GCIPL as a standard parameter to be included in future OCT studies in ETPKU. In contrast, Hopf et al. found no retinal alterations in a small cohort of ETPKU patients (37).

The reduction of GCIPL volume was significant for adults, but not for the pediatric cohort. The observation that neuronal damage increases with age is in line with a report from MRT studies demonstrating increased WMLs with age (56). Nevertheless, there was a trend toward lower GCIPL volume also in our pediatric patients with poorer metabolic control. The metabolic control in our pediatric cohort was, overall, very good. Only 5 out of 31 patients had average Phe values outside the European treatment recommendations. This small number might be one reason why the level of significance was not reached.

Assessing axonal degeneration, we did not observe reduced pRNFL thickness in the adult ETPKU patients, not even in the patients with poorer metabolic control. A reduced pRNFL thickness was also not observed when the temporal quadrant with the particularly vulnerable papillomacular bundles (57) was analyzed separately. This finding is conflicting with the study of Serfozo et al. reporting significantly reduced pRNFL thickness in adult ETPKU correlating with the blood Phe levels (39). In line with the study of Nowak et al. (38), however, we found a significantly reduced pRNFL thickness in pediatric ETPKU patients with average Phe concentrations outside the recommended range.

As expected from other similar indications (34, 35), the changes in OCT parameters reported by us and the other ETPKU studies were small (38–40). Taking into account that PAH deficiency is a rare disease and various factors (e.g., phenotype, metabolic control, and age) might have an impact on retinal neuroaxonal degeneration, a potential bias could be caused by the study-specific characteristics. A possible influence on OCT measures could also result from previously unrecognized ocular or systemic comorbidities. This could be particularly the case in the elderly study participants. However, the risk of influence was minimized by the exclusion criteria, especially since the bias affected both the PKU and HC cohorts. Nevertheless, larger studies are needed to minimize potential bias. OCT is well-suited for standardized data collection (58), enabling multicenter approaches for cross-sectional and longitudinal studies.

Whether the observed retinal changes relate to WML burden remains speculative as MRI scans were not available in any of the OCT studies (38–40), including ours. However, WMLs have repeatedly been described in ETPKU patients with the underlying molecular mechanisms remaining elusive (17–19, 59–63). Intramyelinic edema is primarily thought to be responsible for reversible WMLs (17), but impairment of microstructural development has also been described (59). Likewise, the pathophysiology underlying the described retinal neuroaxonal alterations is not yet understood. Serfozo et al. speculated that alterations in the dopamine levels might contribute to retinal degeneration in ETPKU (39, 40) as it has been suggested for other diseases with perturbations in the dopaminergic system, such as Parkinson's disease (64, 65). Dopamine plays a complex role in visual processing (66, 67), and dopaminergic cells are located mainly in the INL (66–69). An investigation of the INL in ETPKU has been suggested (40), but until now, no study has been available.

Our pediatric cohort showed an increased INL volume, and this effect was particularly influenced by those patients with high current Phe concentrations. In other disorders, INL swelling has been associated with macular edema (70) and/or inflammatory activity (71–74). The pathogenesis of INL swelling in our cohort is ultimately unclear. The INL contains a relevant number of Müller cells involved in retinal environmental homeostasis (75). They play a critical role in the regulation of extracellular space volume, water homeostasis, modulation of inflammatory responses, and contribute to oxidative stress (75, 76). It has been previously suggested, that elevated Phe concentrations can induce oxidative stress (77). Therefore, it could be hypothesized that the increased Phe concentrations lead to oxidative stress that triggers activation of Müller glial cells, resulting in swelling of the INL either through Müller cell hypertrophy or edema. Whether neuroinflammatory processes, as repeatedly discussed in PKU (60), also play a role remains speculation.

In ETPKU, no WMLs have been described below Phe concentrations of 360 μmol/L, data at concentrations between 360 and 600 μmol/L are inconsistent (25, 78, 79). In our cohort, we saw individual ETPKU patients with Phe values <600 μmol/L who had increased INL volume, but there was no overall correlation of the INL volume with the current Phe level. Longitudinal studies are needed to show whether the increase in INL volume will be reversible with improved metabolic control, as has been shown for the WMLs (25, 26). In addition, it remains to be clarified why pediatric but not adult patients showed abnormalities of the INL. One might hypothesize that the developmental switch of retinal cells in the INL may play a role (80).

In conclusion, our data on spectral-domain OCT in PAH deficiency covering the full phenotypic spectrum of the disease provide evidence of retinal neuroaxonal degeneration and INL swelling depending on phenotype, current age, and metabolic control. These findings suggest that OCT is a suitable marker to investigate neurodegeneration in PKU. We propose OCT as a sensitive, reliable, safe, low-burden, and low-cost examination to contribute to the urgent questions of treatment indications and targets in future larger and multicenter studies.

## Data Availability Statement

The original contributions presented in the study are included in the article/supplementary material, further inquiries can be directed to the corresponding author/s.

## Ethics Statement

The studies involving human participants were reviewed and approved by Ethics Committee of the Ludwig-Maximilians-University of Munich, Medical Faculty. Written informed consent to participate in this study was provided by the participant, and/or the participants' legal guardian/next of kin.

## Author Contributions

AL-H designed and conceptualized the study, played a major role in the acquisition of data, analyzed the data, was responsible for the statistical analyses, and drafted the manuscript. JH designed and conceptualized the study, played a major role in the acquisition of data, and drafted the manuscript. EM designed and conceptualized the study and drafted the manuscript. TC, LB, SR-V, KW, KS, and KP were involved in the acquisition of data. All the authors contributed to the final version of the manuscript.

## Funding

This study was supported by a research grant from Nutricia Metabolics. JH is (partially) funded by the German Federal Ministry of Education and Research [Grant Numbers 01ZZ1603[A-D] and 01ZZ1804[A-H] (DIFUTURE)].

## Conflict of Interest

The authors declare that this study received funding from Nutritia Metabolics. The funder was not involved in the study design, collection, analysis, interpretation of data, the writing of this article or the decision to submit it for publication. AL-H has received travel reimbursement from BioMarin and Sobi. KW has received travel reimbursement from Sobi and Amicus Therapeutics, and honoraria for lectures from Sobi and Nutritia Metabolic. KS has received travel reimbursement from Shire and BioMarin. KP has received research funding and/or honoraria for consultancy and/or speaker's bureau activity from BioMarin, Sobi, Dr. Schaer. JH reports a grant for OCT research from the Friedrich-Baur-Stiftung, personal fees, and non-financial support from Merck, Alexion, Novartis, Roche, Santhera, Biogen, Heidelberg Engineering, Sanofi Genzyme, and non-financial support of the Guthy-Jackson Charitable Foundation, all outside the submitted work. EM has received travel reimbursement from Sobi and Dr. Schär and was paid for advisory services from Sobi, APR, and Sanofi-Aventis. The remaining authors declare that the research was conducted in the absence of any commercial or financial relationships that could be construed as a potential conflict of interest.

## Publisher's Note

All claims expressed in this article are solely those of the authors and do not necessarily represent those of their affiliated organizations, or those of the publisher, the editors and the reviewers. Any product that may be evaluated in this article, or claim that may be made by its manufacturer, is not guaranteed or endorsed by the publisher.
